# Sepsis leads to lasting changes in phenotype and function of naïve CD8 T cells

**DOI:** 10.1371/journal.ppat.1011720

**Published:** 2023-10-12

**Authors:** Roger R. Berton, Patrick W. McGonagil, Isaac J. Jensen, Tiffany K. Ybarra, Gail A. Bishop, John T. Harty, Thomas S. Griffith, Vladimir P. Badovinac

**Affiliations:** 1 Interdisciplinary Graduate Program in Immunology, University of Iowa, Iowa City, Iowa, United States of America; 2 Department of Pathology, University of Iowa, Iowa City, Iowa, United States of America; 3 Department of Surgery, University of Iowa, Iowa City, Iowa, United States of America; 4 Department of Microbiology and Immunology, Columbia University Irving Medical Center, New York City, New York, United States of America; 5 Department of Microbiology and Immunology, University of Iowa, Iowa City, Iowa, United States of America; 6 Department of Urology, University of Minnesota, Minneapolis, Minnesota, United States of America; 7 Minneapolis Veterans Affairs Health Care System, Minneapolis, Minnesota, United States of America; University of Utah, UNITED STATES

## Abstract

Sepsis, an amplified immune response to systemic infection, is characterized by a transient cytokine storm followed by chronic immune dysfunction. Consequently, sepsis survivors are highly susceptible to newly introduced infections, suggesting sepsis can influence the function and composition of the naïve CD8 T cell pool and resulting pathogen-induced primary CD8 T cell responses. Here, we explored the extent to which sepsis induces phenotypic and functional changes within the naïve CD8 T cell pool. To interrogate this, the cecal ligation and puncture (CLP) mouse model of polymicrobial sepsis was used. In normal, non-septic mice, we show type-I interferon (IFN I)-mediated signaling plays an important role in driving the phenotypic and functional heterogeneity in the naïve CD8 T cell compartment leading to increased representation of Ly6C^+^ naïve CD8 T cells. In response to viral infection after sepsis resolution, naïve Ly6C^+^ CD8 T cells generated more primary effector and memory CD8 T cells with slower conversion to a central memory CD8 T cell phenotype (Tcm) than Ly6C^-^ naïve CD8 T cells. Importantly, as a potent inducer of cytokine storm and IFN I production, sepsis leads to increased representation of Ly6C^+^ naïve CD8 T cells that maintained their heightened ability to respond (i.e., effector and memory CD8 T cell accumulation and cytokine production) to primary LCMV infection. Lastly, longitudinal analyses of peripheral blood samples obtained from septic patients revealed profound changes in CD8 T cell subset composition and frequency compared to healthy controls. Thus, sepsis has the capacity to alter the composition of naïve CD8 T cells, directly influencing primary CD8 T cell responses to newly introduced infections.

## Author summary

Sepsis is an exaggerated host response to systemic infection that can lead to significant mortality and long-lasting immune dysfunction in survivors. Sepsis-induced immune dysfunction contributes to the increased susceptibility to new infections and increased cancer incidence. These findings suggest naïve CD8 T cells are negatively impacted following a septic event, but the underlying mechanism(s) responsible for naïve CD8 T cell impairment and the consequences for CD8 T cell-mediated pathogen control remain understudied. Naïve CD8 T cells are required for the control of newly encountered pathogens and have been recently appreciated as a functionally and phenotypically heterogenous compartment. The present study defines Type I IFN as a signal which shapes naïve CD8 T cell heterogeneity at homeostasis. Following a septic event, the naïve CD8 T cell compartment undergoes lasting phenotypic and functional changes in mice and humans. Type I IFN signaling along with increased cell cycling in the post-septic environment contribute to this compositional change, which may have direct implications for therapeutic intervention to enhance CD8 T cell function in immunocompromised sepsis survivors.

## Introduction

Sepsis, a dysregulated systemic immune response to an uncontrolled pathogen, affects ~130,000 people daily with a >20% mortality rate worldwide. Within the United States, sepsis accounts for >$20B in economic burden annually [1,2]. Sepsis can be immunologically characterized by an initial transient cytokine storm, followed by a chronic state of immune dysfunction termed immunoparalysis [3,4]. The cytokine storm is composed of both pro- and anti-inflammatory cytokines; where pro-inflammatory cytokines aim to control the systemic pathogen and anti-inflammatory cytokines counterbalance the pro-inflammatory state [5]. While ~75% of patients survive the transient cytokine storm, previously septic individuals have reduced 5-year survival rates relative to non-septic cohorts. Sepsis-induced immunoparalysis is immunologically defined, in-part, by transient lymphopenia and reduced capacity of multiple lymphocyte populations to exert their effector functions properly [6–13]. Consequently, there are now considerable data supporting the idea that the immunoparalysis phase of sepsis encompasses the majority of sepsis-associated deaths [14,15]. Sepsis-induced immunoparalysis increases host susceptibility to both previously and newly encountered infections and cancer incidence [16–19]. Interestingly, sepsis survivors also show reduced susceptibility to autoimmune diseases, highlighting the general sepsis-induced immunologic impairment [20].

Sepsis-induced lymphopenia impacts both naïve and memory CD8 T cells early after sepsis [16,21–23]. CD8 T cells in the circulation and secondary lymphoid organs are most sensitive to the apoptosis-inducing cues generated during sepsis. In contrast, tissue-resident memory (Trm) CD8 T cells, due to their localization inside tissues, are less prone to sepsis-induced deletion and require a more severe septic event for their numerical and functional loss [24]. Naïve CD8 T cells can undergo thymus-independent numerical recovery (i.e., homeostatic proliferation) during the immunoparalysis phase, and the cells that undergo homeostatic proliferation adopt a memory-like phenotype (CD8a^lo^CD11a^hi^CD44^hi^CD49d^-^) [16,22,25]. In addition, not all CD8 T cell TCR-specificities that experience sepsis-induced reductions return to baseline numbers. These features of the post-sepsis CD8 T cell compartment suggest certain TCR clones preferentially recover, resulting in the skewed composition of the TCR repertoire [22]. CD8 T cells that survive the cytokine storm are also less capable of manifesting antigen (Ag)-dependent effector functions and have increased inhibitory receptor (e.g., 2B4, Lag3, and PD-1) expression [19,26,27], which decreases the hosts capacity to control new infections and cancer early after sepsis [18,19]. However, the extent to which sepsis impacts the naïve CD8 T cell compartment and the consequences of these changes during the immunoparalysis phase in sepsis survivors remains largely unknown and understudied.

Naïve CD8 T cells are typically considered to be a functionally and phenotypically homogenous population compared to memory CD8 T cells, and thus their complexity is underappreciated. However, recent reports show naïve CD8 T cells have different functional capacities based on their CD5 expression (i.e., strength of TCR interaction with self-ligands), which can vary within individual TCR-specificities [28–30]. Specifically, CD5^hi^ naïve CD8 T cells expand greater than CD5^lo^ cells following primary and secondary challenges [28]. Delineation of naïve CD8 T cells based on CD5 or Ly6C expression has also shown this compartment is phenotypically heterogeneous, where more CD5^hi^ and Ly6C^+^ cells are CXCR3^+^ and CD103^-^ than their counterparts [31]. Furthermore, at steady-state, Ly6C^+^ naïve CD8 T cells contain a transcriptome that more closely resembles an activated effector, compared to Ly6C^-^ naïve CD8 T cells [31,32]. Therefore, the objective of this study was to address the extent to which sepsis induces phenotypic and functional changes within the heterogenous naïve CD8 T cell pool.

Here, we demonstrate sepsis can markedly impact the phenotypic and functional heterogeneity of naïve CD8 T cells via Type I IFN signaling, leading to an increased representation of Ly6C^+^ naïve CD8 T cells with differential effector responses compared to Ly6C^-^ naïve CD8 T cells. Moreover, post-septic Ly6C^+^ naïve CD8 T cells outcompete and develop into distinct memory CD8 T cells, compared to Ly6C^-^ naïve CD8 T cells, after Ag-specific stimulation. Thus, sepsis has the capacity to markedly alter the composition of naïve CD8 T cells, directly influencing the ability of the host to respond to new infections.

## Results

### Ly6C expression delineates phenotypically distinct naïve CD8 T cells

**Fig 1:**
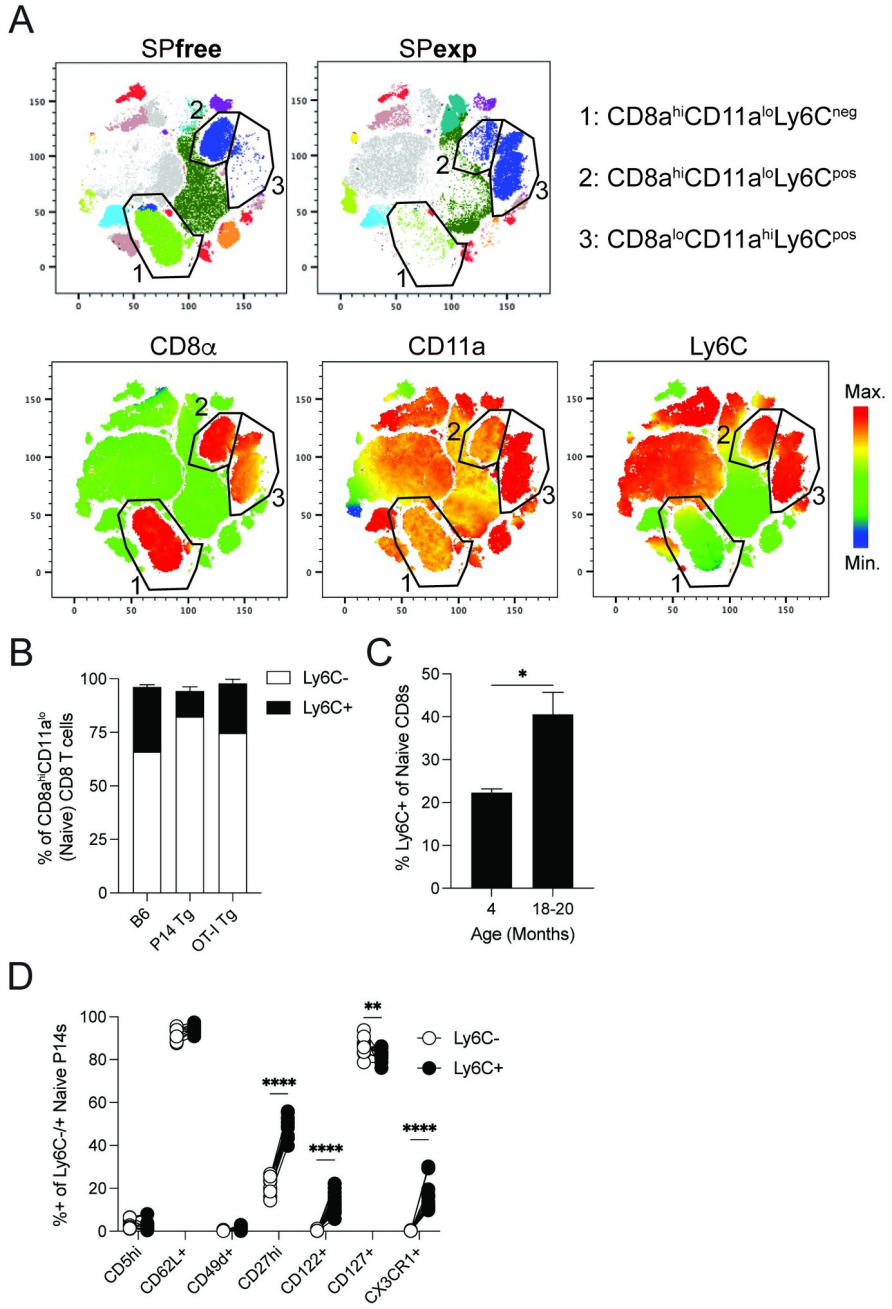
Naïve CD8 T cells are phenotypically heterogenous. (**A**) tSNE analysis of PBL from Specific pathogen free (SPfree) and Specific pathogen experienced (SPexp) mice 5 days post last infection. Sequential infection of SPfree C57BL/6 mice with PR8-IAV, MCMV-Smith, VacV, att. *Lm*., and LCMV-arm in 5 d intervals was used to generate SPexp mice. Top row tSNE plots display clusters enriched in SPfree and SPexp mice and outlined populations define CD8 T cell populations. Bottom row tSNE plots display heatmap overlays of surface markers CD8α, CD11a, and Ly6C used to define CD8 T cell populations 1–3. (**B**) Frequencies of Ly6C^-^ and Ly6C^+^ naïve CD8 T cells in WT, TCR transgenic P14, and OT-I C57BL/6 mice. Prior gating on CD8α^hi^CD11a^lo^ single-cell lymphocytes. (**C**) Ly6C expression on naïve CD8 T cells in 4- and 18–20-month-old WT C57BL/6 mice. (**D**) CD8 T cell related marker expression on Ly6C^-^ and Ly6C^+^ naïve P14 CD8 T cells. Data are representative of at least three independent experiments with at least 5 mice per group. Error bars represent SEM. *p < 0.05, **p < 0.01, ****p < 0.0001.

Heterogeneity among naïve (defined as cognate Ag non-experienced) CD8 T cells has been documented recently based on the expression marker such as CD5 [28,31]. To further define the heterogeneity of naïve CD8 T cells we utilized an unsupervised clustering approach (tSNE) to compare the CD8 T cell compartment in age-matched uninfected specific pathogen-free (SPfree) mice and SPF mice sequentially infected with well-defined BSL-2 pathogens to mimic microbial exposure that host likely encounter throughout the life (Specific Pathogen-experienced; SPexp; Fig 1A) [33]. tSNE clustering defined 3 distinct CD8 T cell populations: populations 1 and 2 are predominant in SPfree mice, while population 3 was highly enriched in SPexp mice (Fig 1A, **upper panels**). Importantly, populations 1 and 2 are bona-fide naïve CD8 T cells since they express higher levels of CD8 and lower CD11a compared to Ag-experienced CD8^lo^CD11a^hi^ population 3 (Figs 1A **and** S1A) [34–37]. Interestingly, naïve CD8 T cell population 2 clusters closer to Ag-experienced CD8 T cells (population 3) due to Ly6C expression (Fig 1A, **lower right panel**). In SPfree inbred B6, OT-I, and P14 mice, 10–20% of naïve CD8 T cells express Ly6C, irrespective of TCR specificity (Fig 1B). The representation of Ly6C^+^CD8^hi^CD11a^lo^ CD8 T cells is also dependent on the age of the B6 host (Fig 1C), as the frequency of Ly6C^+^ naïve CD8 T cells is significantly increased in 18–20-month-old mice compared to 4-month-old mice. Furthermore, >90% of all naïve CD8 T cell express Ly6C in SPexp mice. Finally, to establish whether Ly6C expression demarcates phenotypically distinct naïve CD8 T cells, we examined the expression of CD8 T cell-related molecules on Ly6C^-^ and Ly6C^+^ naïve CD8 T cells (Figs 1D **and** S1C). We found an increased proportion of Ly6C^+^ naïve CD8 T cells expressed CD27, CD122, and CX3CR1, suggesting this subpopulation of naïve CD8 T cells may be poised to readily exert their effector functions, proliferate in response to exogenous cytokines, and/or migrate to infected tissues.

### Ly6C^+^ naïve CD8 T cells generate increased number of effector and memory CD8 T cells with delayed conversion to central memory CD8 T cells

**Fig 2:**
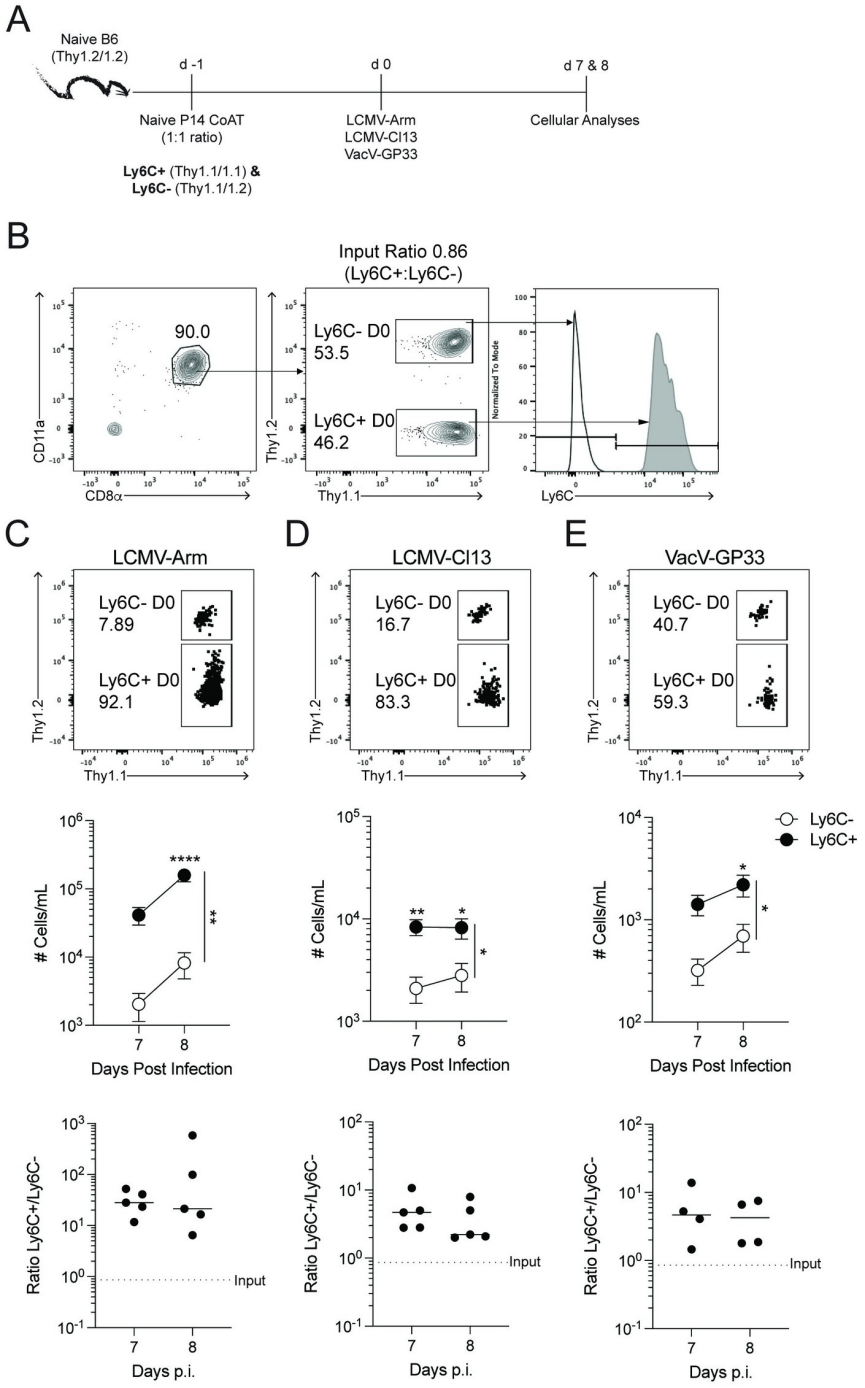
Naïve Ly6C^+^ CD8 T cells have increased accumulation early after viral challenge. (**A**) Experimental design. Thy1.2^+^/1.2^+^ C57BL/6 mice received 5×10^3^ Ly6C^-^ (Thy1.1^+^/1.2^+^) and Ly6C^+^ (Thy1.1^+^/1.1^+^) naïve P14 CD8 T cells followed by an infection with LCMV-Arm, LCMV-Cl13, or VacV-GP33 1 day later via canonical infection routes. Cellular responses were monitored in peripheral blood 7- and 8-days post infections. (**B**) Flow plot example of injected cell master mix. Numbers indicate percentage of cells in each gate. P14 CD8 T cell responses following (**C**) LCMV-Arm, (**D**) LCMV-Cl13, or (**E**) VacV-GP33 infection. Top; Flow plot of transferred cells distinguished based on Thy1 disparity. Numbers are frequency of cells of all P14 CD8 T cells. Middle; number of transferred P14 CD8 T cells per mL of blood 7- and 8-days post infection. Bottom; Ratio of transferred P14 CD8 T cells that were Ly6C^+^ (Thy1.1^+^/1.1^+^) or Ly6C^-^ (Thy1.1^+^/1.2^+^) at the time of co-transfer. Dotted line indicates input ratio of Ly6C^+^:Ly6C^-^ naïve CD8 T cells. Data are representative from at least three independent experiments with at least 5 mice per group. Error bars represent SEM. *p < 0.05, **p < 0.01, ****p < 0.0001.

**Fig 3:**
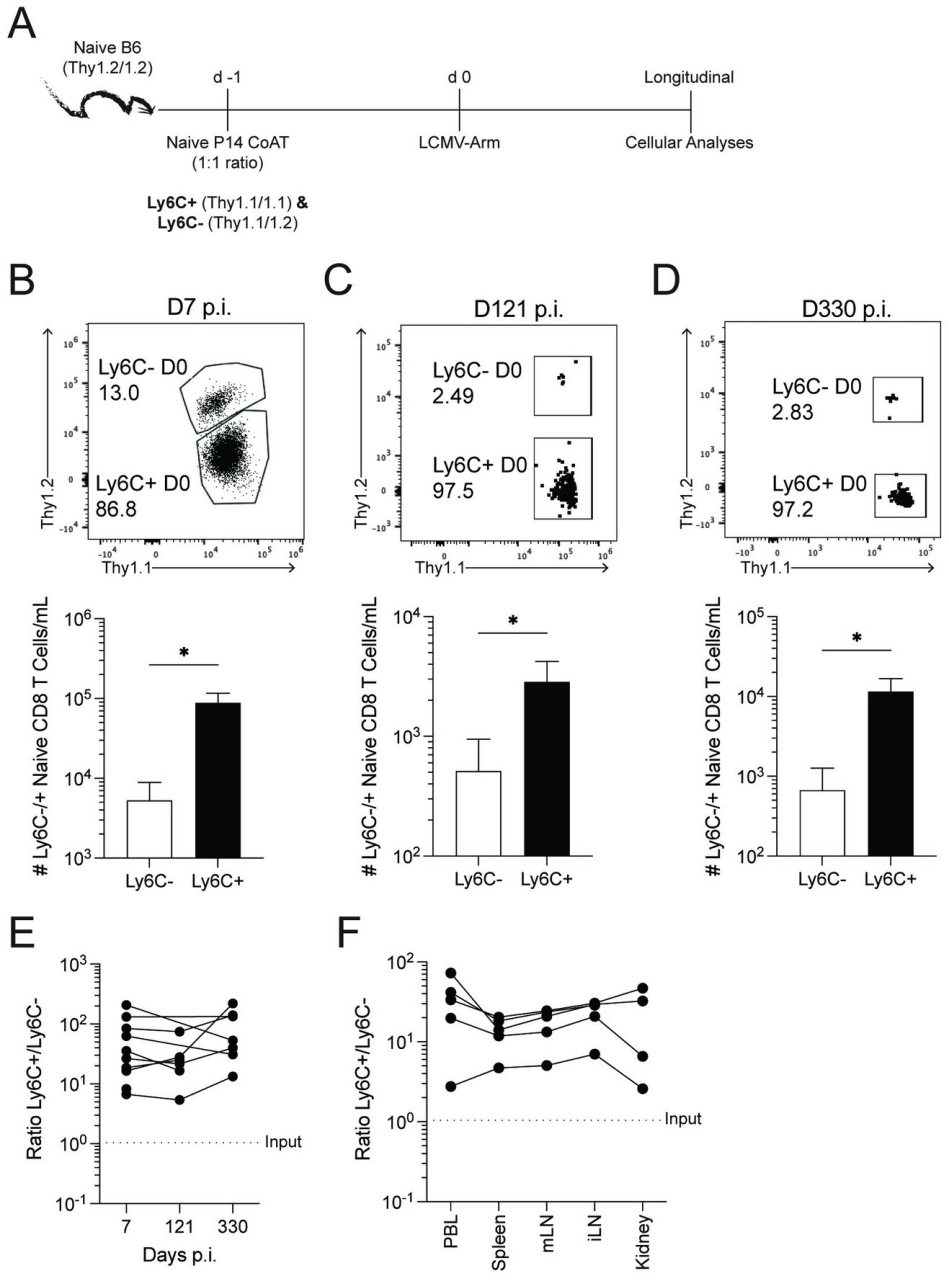
Ly6C^+^ naïve CD8 T cells have increased memory generation potential and tissue seeding. (**A**) Experimental design. Thy1.2^+^/1.2^+^ C57BL/6 mice received 10^4^ Ly6C^-^ (Thy1.1^+^/1.2^+^) and Ly6C^+^ (Thy1.1^+^/1.1^+^) naïve P14 CD8 T cells followed by an infection with LCMV-Arm 1 day later. Cellular responses were monitored in peripheral blood at (**B**) 7-, (**C**) 121-, and (**D**) 330-days post-infection. Top; Flow plot of transferred cells distinguished based on Thy1 disparity. Numbers are frequency of cells of all P14 CD8 T cells. Bottom; numbers of transferred P14 CD8 T cells per mL of blood. (**E**) Kinetic analysis of the ratio of Ly6C^+^:Ly6C^-^ P14 CD8 T cells. Dotted line indicates input ratio of Ly6C^+^:Ly6C^-^ naïve CD8 T cells. (**F**) Ratio of Ly6C^+^:Ly6C^-^ transferred P14 CD8 T cells within tissues 154 days post-infection. Data are representative from at least three independent experiments with at least 5 mice per group. Error bars represent SEM. *p < 0.05.

To test the extent to which Ly6C^±^ naïve CD8 T cells have enhanced effector responses following infection, we co-adoptively transferred equal numbers of TCR-transgenic Ly6C^+^ (Thy1.1/1.1) and Ly6C^-^ (Thy1.1/1.2) naïve P14 CD8 T cells into Thy1.2/1.2 recipient B6 mice 1 day before challenge with several distinct viruses (including LCMV Arm and Clone 13 strains, Vaccinia virus) expressing the GP33 epitope (Fig 2A and 2B). This model minimizes the role of CD8 T cell-extrinsic factors that may impact primary CD8 T cell responses to viral infection(s) because both the Ly6C^+^ and Ly6C^-^ P14 T cells were transferred into the same recipient mice. Irrespective of viral pathogen and route of infection, we found increased proportions and numbers of P14 cells derived from naïve Ly6C^+^ CD8 T cells within the blood 7 and 8 d.p.i. compared to those derived from naïve Ly6C^-^ CD8 T cells (Fig 2C–2E). These findings suggest Ly6C^+^ naïve CD8 T cells have intrinsically enhanced proliferative responses. It is important to note that all effector cells upregulated Ly6C expression regardless of the level of Ly6C expression before viral challenge. To document the durability of the enhanced accumulation from Ly6C^+^ naïve CD8 T cells, we extended our analyses to 330 d.p.i. (Fig 3). Importantly, we observed an increased accumulation of cells derived from Ly6C^+^ P14 CD8 T cells 121 and 330 d.p.i. (Fig 3B–3E). Additionally, the increased accumulation in the circulation was mirrored in secondary lymphoid organs and kidneys 154 d.p.i. (Fig 3F). Collectively, Fig 3 demonstrates Ly6C^+^ naïve CD8 T cells have increased accumulation with similar long-term maintenance compared to Ly6C^-^ naïve CD8 T cells.

**Fig 4:**
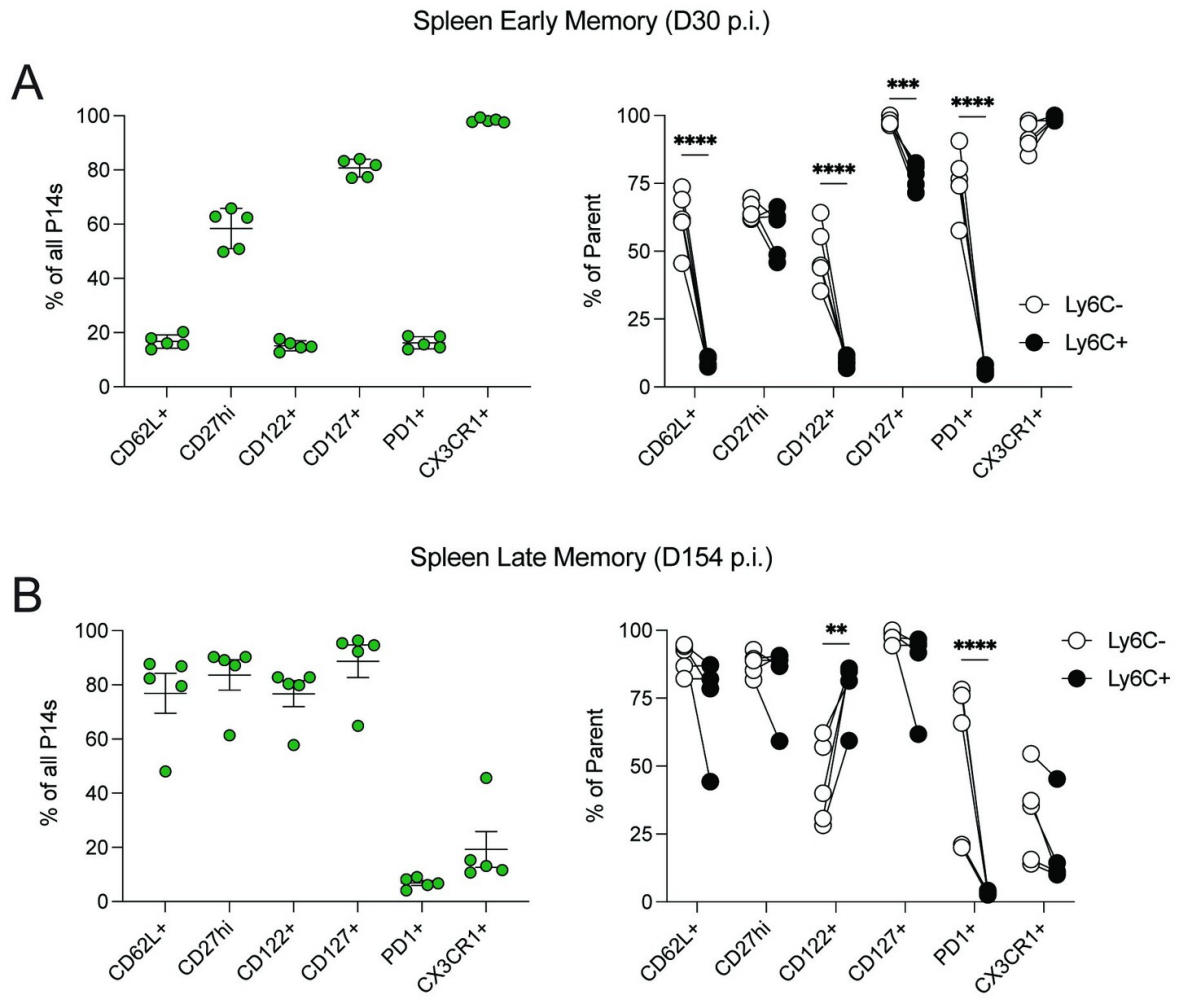
Ly6C distinguishes naïve CD8 T cells with distinct memory cell formation capacity. (**A**) CD8 T cell related marker expression of transferred P14 CD8 T cells 30 days post-infection. Left; Marker expression on P14 CD8 T cells irrespective of Ly6C expression. Right; Expression differences between P14 CD8 T cells that were Ly6C^+^ or Ly6C^-^ at the time of co-transfer. (**B**) CD8 T cell related marker expression on transferred P14 CD8 T cells 154 days post-infection. Left; Marker expression on P14 CD8 T cells irrespective of Ly6C expression. Right; Expression differences between P14 CD8 T cells that were Ly6C^+^ or Ly6C^-^ at the time of co-transfer. Data are representative from at least three independent experiments with at least 5 mice per group. Error bars represent SEM. **p < 0.01, ***p < 0.001 ****p < 0.0001.

Following infection, activated Ag-specific naïve CD8 T cells become a heterogenous memory CD8 T cell population composed of circulating effector and central memory (Tcm) CD8 T cells, and CD8 Trm cells. Additionally, the composition of memory CD8 T cells shifts towards central memory (CD62L^+^) CD8 T cells as time progresses [38–40]. Therefore, we investigated memory formation and phenotypic differences between our two naïve CD8 T cell populations at early (30 d.p.i.) and late (154 d.p.i.) memory time points (Fig 4). As expected, memory CD8 T cell related markers were expressed to varying levels when all transferred P14 CD8 T cells (regardless of Ly6C expression at naïve state) were analyzed at early and late time points (Fig 4A and 4B, **left**). Importantly, the frequency of CD62L^+^ P14 CD8 T cells increased from ~20% at 30 d.p.i. to ~80% at 154 d.p.i. These data suggest the memory P14 CD8 T cells are transitioning to a central memory phenotype (Fig 4A and 4B, **left**). Next, we examined to what extent the Ly6C^-^ or Ly6C^+^ naïve P14 CD8 T cells contribute to the phenotype of the bulk P14 CD8 T cell population at the early and late memory time points, and found the memory cells in the spleen formed from Ly6C^-^ and Ly6C^+^ naïve CD8 T cells were phenotypically distinct (Fig 4A and 4B **right**). Specifically, cells derived from Ly6C^+^ naïve P14 CD8 T cells had decreased CD62L, CD122, CD127, and PD-1 expression at early memory (Fig 4A, **right**). Phenotypic differences at this early memory time point were also observed on P14 CD8 T cells present in the peripheral blood (PBL), mesenteric lymph nodes (mLN), and inguinal lymph nodes (iLN; S2A–S2C Fig). Interestingly, the frequency of CD62L^+^ cells no longer differed at late memory, but differences in the frequency of cells expressing CD122 and PD-1 were maintained (Fig 4B, **right**). Together, these data indicate Ly6C^+^ naïve CD8 T cells generate more effector and memory cells after infection than Ly6C^-^ cells, but naïve Ly6C^-^ cells upon cognate Ag recognition convert to a central memory phenotype (CD62L^+^) faster.

### Type I interferon-mediated signaling induces Ly6C expression on naïve CD8 T cells

**Fig 5:**
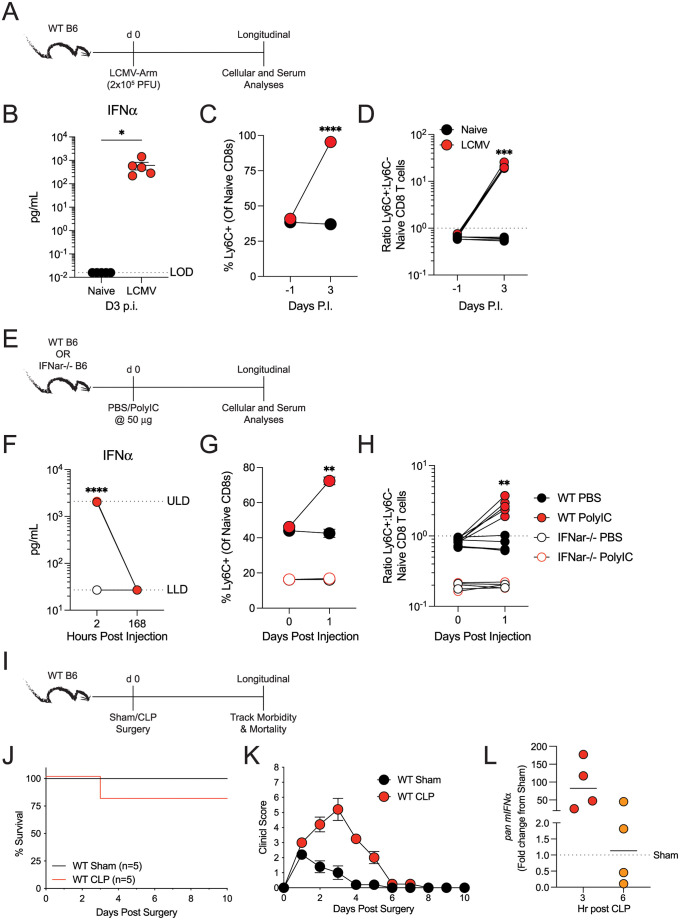
Type I interferon signaling is required for Ly6C expression on naïve CD8 T cells. (**A**) Experimental design. Mice were infected with LCMV-Arm and 3 days later serum and naïve CD8 T cells were analyzed. (**B**) Amount of IFNα (pg/mL) within the serum of naïve and LCMV-Arm infected mice 3 days post-infection. (**C**) Frequency of Ly6C^+^ naïve CD8 T cells prior to and 3 days post-LCMV-Arm infection. (**D**) Ratio of Ly6C^+^:Ly6C^-^ naïve CD8 T cells before and 3 days post-LCMV-Arm infection. (**E**) Experimental Design. WT or *Ifnar*^*-/-*^ C57BL/6 mice were injected with PBS or 50 μg of poly(I:C). At various timepoints after injection, serum and naïve CD8 T cells were analyzed for IFNα and Ly6C expression, respectively. (**F**) Amount of IFNα in serum 2- and 168-hours post-PBS or poly(I:C) injection. (**G**) Frequency of Ly6C^+^ naïve CD8 T cells before and 1 day post-PBS or poly(I:C) injection. (**H**) Ratio of Ly6C^+^:Ly6C^-^ naïve CD8 T cells before and 1-day post-injection. (**I**) Experimental design. Morbidity and mortality were monitored in mice that underwent cecal ligation and puncture (CLP) or sham (control) surgery. (**J**) Mortality and (**K**) morbidity of mice after surgery. (**L**) *Ifnα* message in splenocytes 3- and 6-hours post-surgery. Data are representative from at least three independent experiments with at least 5 mice per group. Error bars represent SEM. *p < 0.05, **p < 0.01, ***p < 0.001 ****p < 0.0001.

Ly6C expression on naïve CD8 T cells can be induced *in vitro* in a dose-dependent manner when stimulated with IFNα or IFNβ [31,32]. Therefore, we sought to determine which *in vivo* scenario upregulated Type I Interferons (IFN) and whether Type I IFN signaling was required for Ly6C expression *in vivo*. LCMV-Arm induces IFNα expression 3 d.p.i. [36]; thus, we first measured Ly6C expression on bona fide naïve CD8 T cells following a LCMV-Arm infection (Fig 5A). As expected, LCMV infection increased IFNα concentration in the serum 3 d.p.i. (Fig 5B). The frequency of Ly6C-expressing naïve CD8 T cells and ratio of Ly6C^+^:Ly6C^-^ naïve CD8 T cells increased in LCMV infected mice 3 d.p.i. (Fig 5C and 5D). To determine if Type I IFN signaling was required to induce Ly6C expression on naïve CD8 T cells, we administered poly(I:C) or PBS to WT and *Ifnar*^*-/-*^ mice and tracked Ly6C expression (Fig 5E). Poly(I:C) is a potent Type I IFN inducer [41], and *Ifnar*^*-/-*^ mice cannot mediate Type I IFN signaling [31]. IFNα concentration in the serum was increased 2 hours post-poly(I:C) administration in both WT and *Ifnar*^*-/-*^ mice (Fig 5F). Importantly, frequencies of Ly6C^+^ naïve CD8 T cells only increased in poly(I:C)-treated WT mice 1 day post-injection, leading to an increased ratio of Ly6C^+^:Ly6C^-^ naïve CD8 T cells (Fig 5G and 5H). Lastly, we asked to what extent polymicrobial sepsis induces Type I IFN message by utilizing the CLP model (Fig 5I). CLP mice had increased mortality and clinical score, compared to sham controls (Fig 5J and 5K). Additionally, we detected increased amounts of *Ifnα* mRNA within the spleen 3 hr post-sepsis induction (Fig 5L). Overall, our data suggest that Ly6C expression on naïve CD8 T cells is controlled by Type I IFNs produced following CLP surgery, raising the possibility that sepsis may alter the composition of the naïve CD8 T cell pool.

### Sepsis alters the composition of naïve CD8 T cells by increasing representation of cells with enhanced function

**Fig 6:**
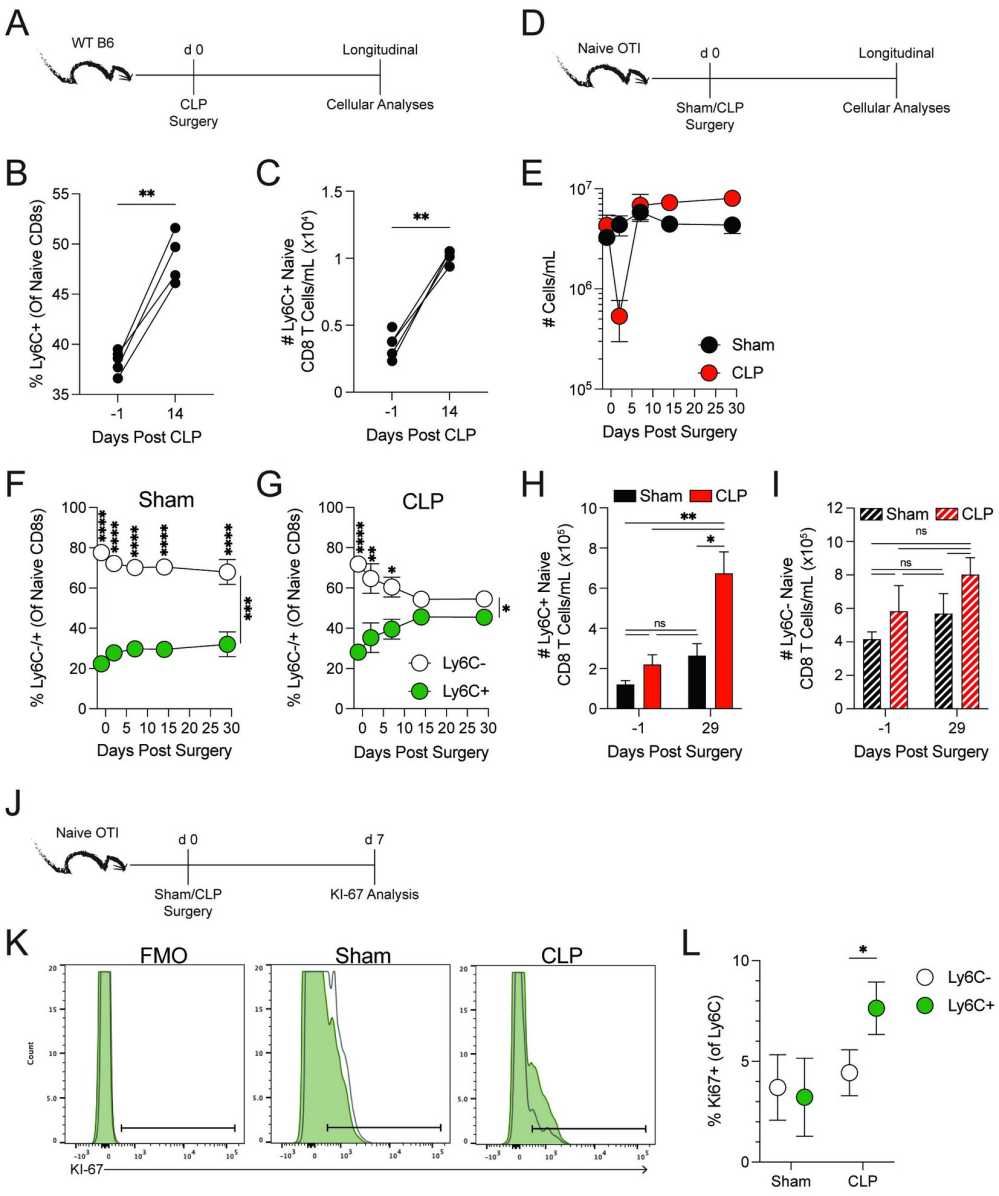
Sepsis has the capacity to influence the composition of naïve CD8 T cell compartment. (**A**) Experimental design. C57BL/6 mice underwent CLP surgery and the expression of Ly6C on naïve CD8 T cells was monitored. (**B**) Frequency and (**C**) number of Ly6C^+^ naïve CD8 T cells 1 day before or 14 days post-CLP surgery. (**D**) Experimental design. OT-I mice underwent sham or CLP surgery and the expression of Ly6C on OT-I cells in the peripheral blood was monitored. (**E**) Number of CD45^+^ leukocytes per mL of blood following surgery. Frequency of Ly6C^+^ and Ly6C^-^ naïve CD8 T cells in (**F**) Sham and (**G**) CLP mice overtime. (**H**) Number of Ly6C^+^ naïve CD8 T cells per mL of blood 1 day before and 29 days post-surgery. (**I**) Number of Ly6C^-^ naïve CD8 T cells per mL of blood 1 day before and 29 days post-surgery. (**J**) Experimental design. OT-I mice underwent sham or CLP surgery, and Ki-67 expression in naïve CD8 T cells was assessed 7 days later. (**K**) Example flow plots and (**L**) frequency of Ki-67 expressing naïve OT-I CD8 T cells 7 days pos-surgery. Data are representative from at least three independent experiments with at least 5 mice per group. Error bars represent SEM. ns: no significance, *p < 0.05, **p < 0.01, ***p < 0.001 ****p < 0.0001.

To determine the effect of increased *Ifna* mRNA following a septic event in naïve CD8 T cell compartment we tracked Ly6C expression following CLP surgery (Fig 6A). CLP mice had increased frequencies and numbers of Ly6C^+^ naïve CD8 T cells 14 days post-surgery (Fig 6B and 6C), which was dependent on the cells being able to receive Type I IFN signals (S3A–S3D Fig). To confirm the increase in Ly6C expression on naïve CD8 T cells was not due to TCR-mediated recognition of Ag present in the fecal matter released during the CLP procedure [42], we utilized OT-I TCR-transgenic mice because naïve CD8 T cells from these mice should not (to our knowledge) be stimulated by gut-derived antigens (Fig 6D). As expected, CLP OT-I mice experience lymphopenia 2 days post-surgery (Fig 6E). Importantly, the septic event increased the frequency and number of Ly6C^+^ naïve CD8 T cells, which began to increase by day 7 post-CLP (Fig 6F–6H). Notably, CLP did not increase the number of Ly6C^-^ naïve CD8 T cells 29 days post-septic induction (Fig 6I). There was also an increased frequency of Ki67^+^ Ly6C^+^ naive CD8 T cells from CLP mice 7 days after surgery, without increasing the frequency of proliferating Ly6C^-^ cells (Fig 6J–6L), suggesting Ly6C^+^ naïve CD8 T cells had increased proliferative capacity. Together, these data show sepsis has the capacity to increase the number of Ly6C^+^ naïve CD8 T cells without altering the Ly6C^-^ fraction. The observed increase of Ly6C^+^ naïve CD8 T cells is likely through both their increased cell cycling, and through Type I IFN-mediated conversion of Ly6C^-^ naïve CD8 T cells to Ly6C^+^ cells. Type I IFN-mediated conversion in the post-septic environment is supported by Ly6C^-^ naïve CD8 T cells having basal Ki67 positivity (~5% Ki67^+^; Fig 6L) without their numerical increase.

We were also curious whether there were any other phenotypic differences between Ly6C^+^ and Ly6C^-^ naïve CD8 T cells following a septic event. As seen in Fig 1D, phenotypic differences between Ly6C± naïve CD8 T cells were maintained in Sham and CLP operated hosts (S3E Fig). Thus, new Ly6C^+^ naïve CD8 T cells produced during sepsis maintain phenotypic differences, compared to Ly6C^-^ cells. These data collectively suggest sepsis survivors have increased naïve CD8 T cells that are impaired in converting to central memory CD8 T cells following activation.

**Fig 7:**
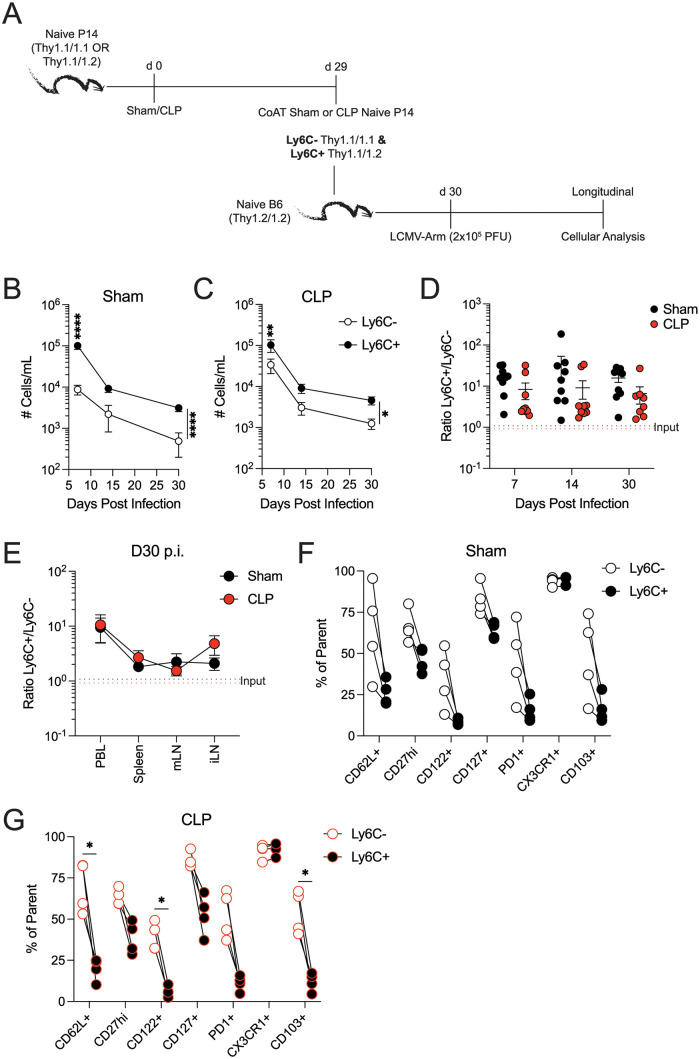
Sepsis-induced Ly6C^+^ naïve CD8 T cells have enhanced accumulation following viral infection. (**A**) Experimental design. Thy1.1^+^/1.1^+^ and Thy1.1^+^/1.2^+^ P14 mice underwent sham or CLP surgery. Spleens were collected 29 days post-surgery to isolate Thy1.1^+^/1.1^+^ Ly6C^-^ and Thy1.1^+^/1.2^+^ Ly6C^+^ naïve P14 CD8 T cells. 10^4^ sham- or CLP-derived Thy1.1^+^/1.1^+^ Ly6C^-^ and Thy1.1^+^/1.2^+^ Ly6C^+^ naïve P14 CD8 T cells were co-transferred into naïve C57BL/6 recipient mice 1 day before LCMV-Arm infection and longitudinally tracked. Number of (**B**) sham- or (**C**) CLP-derived Ly6C^+^ and Ly6C^-^ P14 CD8 T cells in the blood after infection. (**D**) Ratio of Ly6C^+^:Ly6C^-^ descendants following LCMV-Arm infection. (**E**) Ratio of Ly6C^+^:Ly6C^-^ descendants within tissues 30 days post-infection. Black and red dotted lines indicate the input ratio of Ly6C^+^:Ly6C^-^ naïve P14 CD8 T cells derived from sham and CLP donors, respectively. CD8 T cell related marker expression on transferred Ly6C^+^ and Ly6C^-^ P14 CD8 T cells from (**F**) sham or (**G**) CLP-treated donors 30 days post-infection. Data are representative from at least two independent experiments with at least 4 mice per group. Error bars represent SEM. *p < 0.05, **p < 0.01, ****p < 0.0001.

Ly6C^+^ naïve CD8 T cells have increased granzyme B and IFNγ production after *in vitro* stimulation with anti-CD3/CD28 mAb [32]. To elucidate the consequence of the altered naïve CD8 T cell compartment following sepsis we co-adoptively transferred congenic Ly6C^+^ and Ly6C^-^ naïve P14 CD8 T cells obtained from sham or CLP donor mice into naïve recipients 1 day before LCMV-Arm infection (Fig 7A). The naïve P14 cells were harvested from sham and CLP donors 29 d post-surgery, when Ly6C^+^ naïve CD8 T cells are increased in CLP mice. Both the sham- and CLP-derived Ly6C^+^ naïve CD8 T cells had increased accumulation in the blood after viral infection, compared to Ly6C^-^ cells (Fig 7B–7D). The increase of Ly6C^+^-derived naïve CD8 T cells was also observed within the spleen, mLN, and iLN (Fig 7E). Lastly, as seen in Fig 4A, Ly6C expression on naïve CD8 T cells delineates those cells with distinct memory phenotypes regardless of septic status. Specifically, Ly6C^+^ naïve CD8 T cells have decreased memory cells expressing CD62L, CD122, CD127, PD1, and CD103 (a Trm-associated adhesion molecule) within the spleen 30 d.p.i. (Fig 7F and 7G).

**Fig 8:**
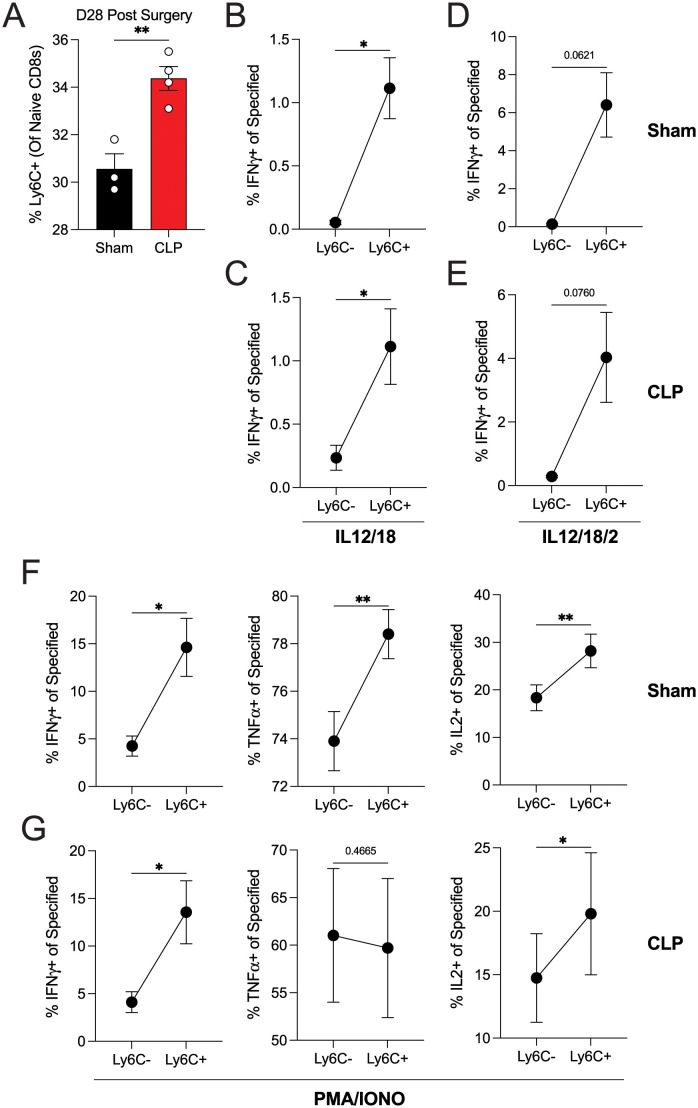
Ly6C^+^ naïve CD8 T cells have enhanced bystander responses following septic event. (**A**) Frequency of Ly6C^+^ naïve CD8 T cells in spleen 28 days post-sham or CLP surgery. Frequency of IFNγ expressing Ly6C^+^ and Ly6C^-^ naïve CD8 T cells 28 days post-surgery after *ex vivo* 5-hour stimulation with (**B-C**) IL-12/IL-18 or (**D-E**) IL-12/IL-18/IL-2. Cytokine production in naïve Ly6C^+^ and Ly6C^-^ CD8 T cells from (**F**) Sham and (**G**) CLP-derived splenocytes following 5 hr *ex vivo* stimulation with PMA/Iono 28 days post-surgery. Left; IFNγ expression, middle; TNFα expression, right; IL2 expression. Data are representative from at least three independent experiments with at least 5 mice per group. Error bars represent SEM. *p < 0.05, **p < 0.01.

In addition to TCR-dependent responses, CD8 T cells can mediate TCR-independent (bystander/innate response) effector functions, such as IFNγ release in response to IL-12/IL-18 [43–45]. Furthermore, sepsis has the capacity to diminish both TCR-dependent and -independent responses [10]. To address if sepsis alters the bystander/innate function of naïve CD8 T cells, we stimulated splenocytes derived from Sham- or CLP-treated mice with IL-12/IL-18 ± IL-2 or PMA/ionomycin for 5 hours (Fig 8). As expected, the frequency of Ly6C^+^ naïve CD8 T cells increased within the spleen 28 d post-CLP (Fig 8A). Notably, the 5-hour *ex vivo* stimulation did not alter Ly6C expression on naïve CD8 T cells. In response to IL-12/IL-18 ± IL-2, Ly6C^+^ naïve CD8 T cells had increased frequency of IFNγ-producing cells, irrespective of the cells coming from sham or CLP mice (Fig 8B–8E). Following PMA/ionomycin stimulation, there were increased frequencies of Ly6C^+^ naïve CD8 T cells from sham mice producing IFNγ, TNFα, or IL-2 (Fig 8F). By comparison, Ly6C^+^ naïve cells from CLP mice had increased frequencies of IFNγ and IL-2, but not TNFα, producers (Fig 8G). Importantly, when comparing cytokine production on a per cell basis between Sham- and CLP-derived Ly6C^+^ naïve CD8 T cells we observed decreased effector cytokines in CLP-derived cells, compared to Sham-derived cells (S4 Fig), suggesting sepsis influences naïve CD8 T cell TCR-independent function. Together, Figs 7, 8 and S4 show sepsis has the capacity to increase the number of Ly6C^+^ naïve CD8 T cells, which have increased TCR-independent and -dependent function and decreased Tcm conversion compared to CLP-derived Ly6C^-^ cells, albeit less than Ly6C^+^ naïve CD8 T cells from Sham hosts. While these results are likely context dependent, the increase in Ly6C^+^ naïve CD8 T cells following sepsis may contribute to the decreased 5-year survival of sepsis patients compared to healthy controls [16].

### Naïve CD8 T cells from septic patients undergo lasting phenotypic changes

**Fig 9:**
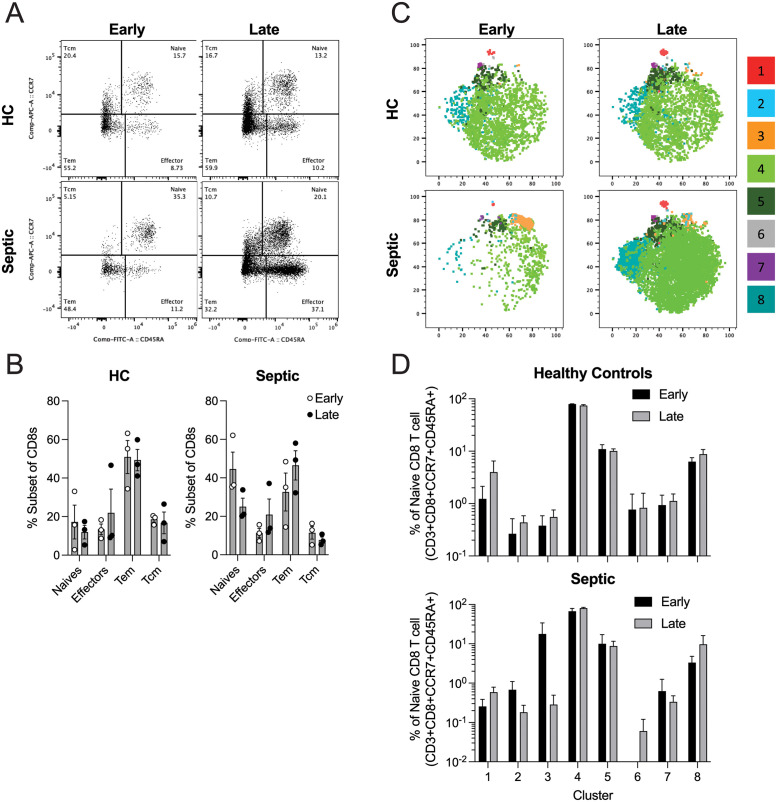
Human naïve CD8 T cells undergo phenotypic changes following septic event. (**A**) Example flow plots of human naïve (CD3^+^CD8^+^CCR7^+^CD45RA^+^), effector (CD3^+^CD8^+^CCR7^-^CD45RA^+^), effector memory (Tem; CD3^+^CD8^+^CCR7^-^CD45RA^-^), and central memory (Tcm; CD3^+^CD8^+^CCR7^+^CD45RA^-^) CD8 T cells from healthy control (HC) and septic patients at early (within 24 hr of admission) and late (≥22 days post-initial blood collection) blood draws. The percentage of cells in each quadrant is indicated. (**B**) Summary data of CD8 T cell subset changes in HC and septic patients at early and late blood draws. (**C**) tSNE analysis of naïve CD8 T cells from HC and septic patients at early and late blood collections. (**D**) Frequency of tSNE defined populations in HC and septic patients at early vs. late collection. Data are representative from at least two independent experiments with 3 subjects per group. Error bars represent SEM.

Lastly, we aimed to begin to establish to what extent naïve CD8 T cells from septic patients differ from non-septic control patients (Fig 9). To this end, we collected longitudinal blood samples from healthy controls (HC) and septic patients at two timepoints separated by 22–28 days. Importantly, early (first) blood collection was within the first 24 hours of ICU admission for septic individuals. The CD8 T cell compartment from the septic hosts was altered when comparing the early and late timepoints. Specifically, the frequency of naïve CD8 T cells decreased and effector/effector memory CD8 T cells increased, which was not observed in HC (Fig 9A and 9B). To establish if naïve CD8 T cells undergo phenotypic changes in septic patients we utilized tSNE analyses on flow cytometric data of naive CD8 T cells from HC and septic patients. Specifically, thawed lymphocytes were stained with mAb against CD4, CD14, CD19 (dump channel), CD8, CD3, CD45RA, CCR7, CD62L, CD127, CX3CR1, Thy1, CD28, KLRG1, CD27, CD57, CD103, and CXCR3. Early septic samples showed a global decrease of the naïve CD8 T cell compartment, compared to healthy controls, with an accumulation of cluster 3 in septic samples (Fig 9C, **top and bottom left**). Notably, cluster 3 was only present in early septic samples. Moreover, the naïve CD8 T cell compartment was restored in the septic individuals at the late blood collection time point (~28 d post-ICU admission; Fig 9C). Furthermore, the naïve CD8 T cell compartment in the sepsis patients underwent phenotypic changes in clusters 2, 3, 6, 7, and 8 from early to late blood draws, which was not observed in the HC samples (Fig 9D). Thus, sepsis has capacity to markedly alter the composition of naïve CD8 T cells directly influencing the ability of the host to respond to new infections. These results could have direct implications on therapeutic intervention to enhance CD8 T cell function in immunocompromised sepsis survivors.

## Discussion

In the present study, we demonstrate that the phenotypic and functional heterogeneity of naïve CD8 T cells is, in part, driven by Type I IFN signaling. Specifically, acute infection-induced Type I IFNs lead to an increased frequency of Ly6C^+^ naïve CD8 T cells. At homeostasis, Ly6C^+^ naïve CD8 T cells display an effector-like phenotype, expressing higher levels of CD27 and CX3CR1. Moreover, Ly6C^+^ naïve CD8 T cells have increased *Eomes* and *Tbx21* transcript expression compared to Ly6C^-^ naïve CD8 T cells [31,32]. We also show Ly6C^+^ naïve CD8 T cells outcompete Ly6C^-^ naïve CD8 T cells following antigenic stimulation, which compliments the effector poised phenotype, but the ability to form Tcm is delayed in Ly6C^+^ naïve CD8 T cells. Importantly, sepsis has the capacity to alter the composition of the CD8 T cell compartment by increasing the number of effector-like Ly6C^+^ naïve CD8 T cells. Mechanistically, sepsis-induced Type I IFNs, in combination with enhanced proliferation in the post-septic environment, increases the number of Ly6C^+^ naïve CD8 T cells which maintained their delayed Tcm formation upon subsequent challenge.

There are several implications of the present study that are important for our understanding of the naïve CD8 T cell pool, and the changes that occur in this subpopulation of immune cells following a septic event. Among these is understanding the effector functions naïve CD8 T cells can exert when responding to an invading pathogen. Our data show Ly6C expression can be utilized to identify naïve CD8 T cells with differential accumulation and tissue seeding capacity. However, it remains to be determined the extent to which Ly6C interactions, during naïve CD8 T cell activation, are contributing to differential responses. Pretreatment of CD8 T cells with Type I IFN enhances cell survival, expansion, and cytotoxicity [46–48], but whether this is due to increased Ly6C signaling in response to Type I IFN-induced Ly6C expression has not been interrogated. Moreover, pretreatment of Tcm CD8 T cells with Ly6C blocking mAb can limit lymph node entry 60 min post transfer [49]. The decreased expansion of Ly6C^-^ naïve CD8 T cells may be due to reduced initial lymph node seeding, albeit hosts were infected 24 hours post-cell transfer making this possibility less likely. Importantly, Ly6C can label preprogramed effector-like naïve CD8 T cells, something which has been used to segregate monocytes with contrasting functions.

Establishing the phenotypic and functional transition of naïve CD8 T cells to memory subset differentiation is of critical importance. Following a primary infection, the responding naïve CD8 T cells form a variety of memory CD8 T cell subsets [50–54]. Upon reinfection CD8 Tcm undergo rapid proliferation to give rise to secondary effector CD8 T cells, which utilize their cytotoxic functions to eliminate infecting pathogens [50,55–57]. The data presented in this study demonstrate Ly6C expression can delineate naïve CD8 T cells with altered capacities for forming Tcm. Specifically, our data suggest Ly6C^-^ naïve CD8 T cells are an important subset to target for immunotherapies due to their enhanced ability to quickly form Tcm. It is important to note that secondary responses between Tcm populations formed from Ly6C^+^ vs. Ly6C^-^ naïve CD8 T cells have yet to be interrogated. Additionally, Trm cells are an important memory subset due to their sensing and alarm functions that rapidly recalls immune cells to the site of infection [58–60], and the propensity of Ly6C^+^ and Ly6C^-^ naïve CD8 T cell subsets to form Trm also needs further investigation.

While the pool of Ly6C^+^ naïve CD8 T cells is established in newborn mice [31], we demonstrate the number of Ly6C^+^ naïve CD8 T cells increases following acute infection (including sepsis). The observed increase in Ly6C^+^ naïve CD8 T cells during a septic event is likely a consequence of Type I IFN production during the sepsis-induced cytokine storm, and due to increased proliferation in the post-septic environment. Additionally, we show the increase in Ly6C^+^ naïve CD8 T cells are irrespective of antigen-specificity as both polyclonal and TCR-transgenic (OVA-specific) naïve CD8 T cells undergo similar compositional changes following CLP. Importantly, Ly6C^+^ naïve CD8 T cells derived from septic hosts maintained their delayed Tcm formation capacity following viral challenge. Although we show sepsis-derived Ly6C^+^ naïve CD8 T cells still maintained altered functions, the consequence of having increased numbers of Ly6C^+^ naïve CD8 T cells need further investigation to understand which aspect(s) negatively influence host responses to infection after sepsis. We hypothesize that having increased effector-poised naïve CD8 T cells would create a host that could overly responds to a new infecting pathogen, making the septic survivor more susceptible to enhanced immunopathology. However, the consequences of the sepsis-induced changes are complex, as the outcome of the newly infected septic survivor may depend on the timing of the newly introduced pathogen and/or the type of pathogen, which can be further complicated by differences in host genetics. Importantly, as a proof-of-concept we show sepsis has the capacity to change the phenotype of naïve CD8 T cell pool in humans. Overall, we show the naïve CD8 T cell compartment in sepsis survivors is profoundly altered leaving hosts with cells that may have reduced memory formation potential. Elucidating the consequence of these changes in septic patients cannot be easily interrogated because of the variability in the human population. It would be interesting to know if any population shifts observed correlated with the outcome of the septic survivor. Importantly, the changes observed in sepsis patients provides a proof-of-concept to our findings that utilize various mouse models. Notably, the number of Ly6C^+^ naïve CD8 T cells increases with age suggesting elderly individuals (a substantial population affected by sepsis [61,62]) may have worse outcomes during future infection than young septic survivors. Thus, it is relevant to consider strategies that mitigate the increase of Ly6C^+^ naïve CD8 T cell pool, such as Type I IFN blockade. Alternatively, establishing those signals that promote an increase in Ly6C^-^ naïve CD8 T cells may be clinically beneficial.

Our characterization of how the naïve CD8 T cell compartment is altered during sepsis demonstrates this compartment undergoes lasting changes, which has consequences on immunity to new infections. The implications of these alterations extend beyond sepsis, and they can also reshape our understanding of immune responses following recent Type I IFN production. Investigating how the naïve CD8 T cell compartment in an immune-experienced host is positioned before and after a septic event may help elucidate if their susceptibility to sepsis is due, in part, to an altered naïve CD8 T cell compartment [63]. Further understanding the signals contributing to the heterogeneity in the naïve CD8 T cell compartment can establish the precise, but complex, early requirements to poise an activating cell to become distinct memory subsets, which has vast therapeutic potential. These lines of investigation would be highly instructive for understanding how naïve CD8 T cells can be preprogrammed for therapeutic interventions to enhance CD8 T cell function.

## Materials and methods

### Ethics statement

Experimental procedures using mice were approved by University of Iowa Animal Care and Use Committee under Animal Care and Use Review, protocol #9101915, and housed under BSL-1 (uninfected and sham/CLP-treated mice) and BSL-2 (infected mice) conditions. All experiments followed Office of Laboratory Animal Welfare guidelines and Public Health Service Policy on Human Care and Use of Laboratory Animals. Experimental animals were humanely euthanized by cervical dislocation.

### Mice and infections

Inbred C57BL/6 mice (wild-type, Thy1.2/1.2) were bred at the University of Iowa. *Ifnar*^*-/-*^ C57BL/6 mice were gifted by Dr. Wendy Maury (University of Iowa). Mice were maintained in the animal facilities at the University of Iowa at the appropriate biosafety level. Both male and female mice >6 weeks old were used in experiments, yielding similar results in both sexes. Specific pathogen-experienced (SPexp) mice were generated as previously described [63]. Briefly, specific pathogen-free (SPfree) mice were sequentially infection with 10^3^ plaque forming units (PFU) influenza A virus A/PuertoRico/8/34 (PR8; H1N1) intranasally (i.n.), followed by 10^5^ PFU MCMV-Smith (or MCMV-K181) intraperitoneally (i.p.), 10^6^ PFU Vaccinia virus i.p., 10^7^ colony forming units (CFU) attenuated *Listeria monocytogenes* (*Lm*.; strain DPL1942) i.v., and 2 × 10^5^ PFU LCMV-Armstrong (LCMV-Arm) i.p. at 5-day intervals. Age matched SPfree mice served as controls. For single pathogen-infected mice, SPfree mice were infected with 2 × 10^5^ PFU of LCMV-Arm i.p., 2 × 10^6^ PFU of LCMV-Cl13 i.v., or 1 × 10^9^ PFU of VacV expressing GP33 (VacV-GP33) intradermally (i.d.). For mice receiving poly(I:C) injections, poly(I:C) was administered at 50 μg per mouse i.p. and control mice received PBS.

### Cell isolation

Peripheral blood was collected retro-orbitally from isoflurane anesthetized mice. Single-cell suspensions from spleen, lymph nodes, and kidneys were generated after mashing tissues through 70 μM cell strainer without enzymatic digestion. ACK lysis buffer was used for red blood cell lysis of PBL and spleen samples.

### Fluorescent labeling, and flow cytometric analysis

Flow cytometry data was acquired on a Cytek Aurora (Cytek, Bethesda, MD) and analyzed with FlowJo software (Tree Star Inc., Ashland, OR). FloJo software was also used for FlowSOM and tSNE analyses. To determine expression of cell surface molecules, mAb were incubated at 4°C for 20–30 min and cells were subsequently fixed for 10 min using Cytofix/Cytoperm Solution (BD Biosciences). In some instances, fixation was followed by incubation with mAb for an additional 20 min to detect intracellular proteins. For Ki-67 analysis, a FoxP3 staining, fixation, and permeabilization set (eBiosciences) was used. The following mAb clones were used: CD8a (53–6.7; eBioscience), Thy1.1 (HIS51; eBioscience), Thy1.2 (53–2.1; eBioscience), CD11a (M17/4; Biolegend), Ly6C (HK1.4; Biolegend), CD127 (eBioSB/199; eBioscience), CD62L (MEL-14; eBioscience), CD122 (TIM-b1; eBioscience), PD1 (J43; eBioscience), CD27 (LG.7F9; eBioscience), CX3CR1 (SA011F11; Biolegend), CD5 (53–7.3; Biolegend), CD49d (R1-2; Biolegend) IFNγ (XMG1.2; eBioscience), TNFα (MP6-XT22; eBioscience), IL-2 (JES6-5H4; eBioscience). The following mAb clones were used for staining patient samples: CD4 (A161A1; Biolegend), CD14 (61D3; Tonbo), CD19 (HIB19; Tonbo), CD45RA (HI100; Tonbo), CCR7 (G043H7; Biolegend), CD8 (HIT8a; Biolegend), CD3 (Hit3a; Tonbo), CD62L (DREG-56; Biolegend), CD127 (A019D5; Biolegend), CX3CR1 (2A9-1; Biolegend), Thy1 (5E10; Biolegend), CD28 (CD28.2; Biolegend), KLRG1 (2F1/KLRG1; Biolegend), CD27 (LG.3A10; Biolegend), CD57 (QA17A04; Biolegend), CD103 (Ber-ACT8; Biolegend), CXCR3 (G025H7; Biolegend).

### Adoptive transfers

To interrogate functional differences between naïve CD8 T cells *in vivo*, Thy-disparate Ly6C^-^ and Ly6C^+^ TCR-Tg naïve P14 CD8 T cells were adoptively transferred into recipient mice at a 1:1 ratio (5–10 x 10^3^ of each sort-purified Ly6C^-/+^ naïve P14 CD8 T cells), followed a day later by infection with LCMV-Arm, LCMV-Cl13, or VacV-GP33. Importantly, a 1:1 ratio of Ly6C^-^:Ly6C^+^ naïve CD8 T cells was confirmed via flow cytometry of injected cells.

### ELISA

IFNα ELISA (Thermofisher) of serum were performed according to the manufacturer’s instructions.

### RT-qPCR

Total RNA was isolated from whole spleen using the RNeasy Plus Mini Kit (Qiagen), according to the manufacturer’s protocol. cDNA was then prepared from RNA using the SuperScript III First-Strand Synthesis SuperMix kit (Invitrogen). Quantitative real-time PCR was performed in triplicate samples with the PowerSYBR Green PCR master mix (ThermoFisher Scientific). The relative expression of *panIFNα* was calculated using the ΔΔCT method. The mouse gene-specific PCR primers are used as follows: *panIFNα* forward primer: 5’ CCTGAGAAGAGAAGAAACACAGCC3’, *panIFNα* reverse primer: 5’GGCTCTCCAGACTTTCTGCTCTG3’; *HPRT* forward primer: 5’TGGATACAGGCCAGACTTTGTT3’, *HPRT* reverse primer: 5’CAGATTCAACTTGCGCTCATC3’.

### Cecal ligation and puncture

Sepsis was induced by cecal ligation and puncture (CLP) [64]. Briefly, mice were anesthetized with ketamine/xylazine, the abdomen was disinfected with Betadine (Purdue Products), and a midline incision was made. Thereafter, the distal third of the cecum was ligated with Perma-Hand Silk (Ethicon), punctured once using a 25-gauge needle, and a small amount of fecal matter was extruded out of puncture. The cecum was then returned to the abdomen, the peritoneum was closed with 641G Perma-Hand Silk (Ethicon). Bupivacaine (Hospira) was then administered at incision site, and skin was closed using surgical Vetbond (3M). Directly following surgery, 1 mL of PBS was administered s.c. to provide post-surgery fluid resuscitation and flunixin meglumine (Phoenix) was administered for post-operative analgesia. Sham mice underwent an identical laparotomy surgical procedures, excluding CLP.

### Sepsis severity evaluation

Clinical signs of sepsis were evaluated and used for scoring disease severity. Clinical scores were assessed by ascending morbidity scale [63,65]. Grooming: 0, (Normal); 1, fur that has lost shine/become matte (Dusty); 2, fur becomes erect or bristling (Ruffled). Mobility: 0, mobile without stimulation (Normal); 1, mice are less responsive/mobile to stimuli (Reduced); 2, mice are unresponsive to stimuli (Immobile). Body Position: 0, body is fully extended (Normal); 1, back is arched/curved (Hunched); 2, laying on side at rest (On side). Weight loss: 0, Due to minimal weight loss that occurs in sham control mice, weight loss has been adjusted to allow for surgery-specific weight loss to be mitigated (<10%); 1, moderate weight loss (10–15%); 2, severe weight loss (>15%). After giving one score for each category, the sum of all categories indicates disease score. Importantly, dead mice are given highest score (8) on the day of death, and thereafter removed from scoring. Healthy scores range from 0–2; moderate disease scores range from 3–5; and severe disease scores range from 6–8.

### Ex vivo stimulation

To assess bystander responses *ex vivo*, bulk splenocyte single-cell suspensions from sham and CLP mice were stimulated for 5 hours with IL-12/IL-18 (10 ng/mL each) in the presence or absence of IL-2 (50 ng/mL), or with PMA/Ionomycin (50 ng/mL each). Additionally, Brefeldin A (GolgiPlug, BD Biosciences) was added for the final hour or the full stimulation with cytokines or PMA/Ionomycin, respectively.

### Institutional setting and IRB approval

Patients were recruited at the University of Iowa Hospitals and Clinics, an 811-bed academic tertiary care center. Blood sample acquisition, patient data collection, and analysis were approved by the University of Iowa Institutional Review Board (ID #201804822). Written consent was obtained from patients or their legally authorized representatives.

### Sepsis patient selection and data collection

Subjects 18 years of age or older meeting Sepsis-3 criteria for sepsis or septic shock secondary to intra-abdominal infection, soft tissue infection, bloodstream infection, or pneumonia were enrolled. Exclusion criteria were infection requiring antibiotics in the past month, hospitalization for infection in the past year, and chemotherapy or radiation within the past year were excluded. EDTA-treated blood samples were collected within 24 hours of presentation and at regular intervals over the subsequent 30 days.

### Healthy control patient selection and data collection

Healthy volunteers 25–80 years of age were recruited from University of Iowa faculty, staff, and graduate/professional students. Exclusion criteria were signs or symptoms of active infections, infection requiring antibiotics within the past month, infection requiring hospitalization in the past year, and chemotherapy or radiation in the past year. EDTA-treated blood samples were collected at an initial visit to our research clinic and a subsequent visit approximately 30 days later.

### Human cell isolation and cryopreservation

Human cell isolation was performed using ACK red cell lysis per previously described methodology [5]. After counting cells, they were resuspended in cell freeze media (90%FCS [Hyclone] 10%DMSO [Fischer Scientific]). Cells were then stored at –80°C until use. When used *in vitro*, peripheral blood leukocytes were rapidly thawed and placed into warmed complete media. Cells were then washed three times with warmed media and aggregates filtered prior to use.

### Statistical analysis

Data were analyzed with Prism 9 GraphPad software using two-tailed Student t-test (for two individual groups, pairing was used for samples that came from the same animal, if unequal variance Mann-Whitney U test was used), one-way ANOVA with Bonferroni post-hoc test (for >2 individual groups, if unequal variance Kruskal-Wallis with Dunn’s post-hoc test was used), two-way ANOVA (for multiparametric analysis of two or more individual groups, pairing was used for samples that came from the same animal), and Kaplan-Meier survival curves with log-rank (Mantel-Cox) tests with a confidence interval of >95% to determine significance (*p < 0.05, **p < 0.01, ***p < 0.001, ****p < 0.0001). Data are presented as standard error of the mean.

## Supporting information

S1 Fig Flow cytometry examples. (**A**) Gating example of naïve (CD8α^hi^CD11a^lo^) and Ag-experienced (CD8α^lo^CD11a^hi^) CD8 T cells in age-matched SPfree and SPexp mice 5 days post-last infection. Pregated on CD3^+^CD8α^+^ single-cell lymphocytes. Numbers indicate the frequency of naïve and Ag-experienced CD8 T cells. (**B**) Flow cytometry plot of Ly6C expression on naïve CD8 T cells in WT C57BL/6 mice. Numbers indicate the frequency of Ly6C^-^ and Ly6C^+^ of naïve CD8 T cells. (**C**) Gating example of CD8 T cell related marker expression on Ly6C^-^ and Ly6C^+^ naïve P14 CD8 T cells. Data are representative from at least three independent experiments with at least 5 mice per group. Error bars represent SEM.
(EPS)

S2 Fig Naïve CD8 T cells form memory cells with distinct phenotypes within tissues. Frequency of marker positive Ly6C^-^ and Ly6C^+^ P14 CD8 T cells 30 days post LCMV-Arm infection in (**A**) PBL, (**B**) mesenteric LN (mLN), and (**C**) inguinal LN [66]. Data are representative from at least three independent experiments with at least 5 mice per group. Error bars represent SEM. *p < 0.05, **p < 0.01, ****p < 0.0001.
(EPS)

S3 Fig Type I IFN signaling is required for altered naïve CD8 T cell compartment following sepsis. (**A**) Experimental design. WT and *Ifnar*^*-/-*^ mice underwent sham or CLP surgery, and the expression of Ly6C on naïve CD8 T cells was monitored. Frequency of Ly6C^-/+^ naïve CD8 T cells in (**B**) sham and (**C**) CLP operated *Ifnar*^*-/-*^ mice. (**D**) Number of Ly6C^+^ naïve CD8 T cells per mL of blood 1 day before and 14 days post-CLP in WT and *Ifnar*^*-/-*^ mice. (**E**) CD8 T cell related marker expression on Ly6C^-^ and Ly6C^+^ naïve CD8 T cells 20 days post-sham/CLP surgery. Data are representative of at least two independent experiments with at least 5 mice per group. Error bars represent SEM. ****p < 0.0001.
(EPS)

S4 Fig Diminished cytokine production in Ly6C^+^ naïve CD8 T cells, compared to Sham-derived Ly6C^+^ naïve CD8 T cells. (**A**) gMFI of IFNγ in Ly6C^+^ naïve CD8 T cells derived from mice 28 days post-sham or CLP surgery after *ex vivo* 5-hour stimulation with IL-12/IL-18/IL-2. (**B**) IFNγ, TNFα, and IL-2 gMFI of Ly6C^+^ naïve CD8 T cells from mice 28 days post-sham or CLP surgery after *ex vivo* 5-hour stimulation with PMA/Iono. Data are representative from at least three independent experiments with at least 5 mice per group. Error bars represent SEM. Numbers indicate p values.
(EPS)
